# The role of various physiological and bioelectrical parameters for estimating the weight status in infants and juveniles cohort from the Southern Cuba region: a machine learning study

**DOI:** 10.1186/s12887-024-04789-w

**Published:** 2024-05-06

**Authors:** Taira Batista Luna, Jose Luis García Bello, Agustín Garzón Carbonell, Ana de la Caridad Román Montoya, Alcibíades Lara Lafargue, Héctor Manuel Camué Ciria, Yohandys A. Zulueta

**Affiliations:** 1 Autonomous University of Santo Domingo (UASD), UASD Nagua Center, Santo Domingo, Dominican Republic; 2 Autonomous University of Santo Domingo (UASD), San Francisco de Macorís Campus, Santo Domingo, Dominican Republic; 3 National Center for Applied Electromagnetism (CNEA), Universidad de Oriente CP 90500, Santiago de Cuba, Cuba; 4 Departamento de Física, Facultad de Ciencias Naturales y Exactas, Universidad de Oriente, Santiago de Cuba, CP 90500 CP Cuba

**Keywords:** Bioimpedance, Machine learning, Biomedical parameters, Fat-free mass, Body mass index

## Abstract

**Objective:**

The search for other indicators to assess the weight status of individuals is important as it may provide more accurate information and assist in personalized medicine.This work is aimed to develop a machine learning predictions of weigh status derived from bioimpedance measurements and other physical parameters of healthy infant juvenile cohort from the Southern Cuba Region, Santiago de Cuba.

**Methods:**

The volunteers were selected between 2002 and 2008, ranging in age between 2 and 18 years old. In total, 393 female and male infant and juvenile individuals are studied. The bioimpedance parameters are obtained by measuring standard tetrapolar whole-body configuration. A classification model are performed, followed by a prediction of other bioparameters influencing the weight status.

**Results:**

The results obtained from the classification model indicate that fat-free mass, reactance, and corrected resistance primarily influence the weight status of the studied population. Specifically, the regression model demonstrates that other bioparameters derived from impedance measurements can be highly accurate in estimating weight status.

**Conclusion:**

The classification and regression predictive models developed in this work are of the great importance for accessing to the weigh status with high accuracy of younger individuals at the Oncological Hospital in Santiago de Cuba, Cuba.

## Introduction

According to World Health Organization (WHO), the body mass index (BMI) quantifies the weight status of individuals. There are various classes differentiating the weight status: underweight (BMI < 18.49 Kg/m^2^), normal weight (BMI 18.50–24.99 Kg/m^2^), overweight (BMI 25.0–29.99 Kg/m^2^), class I obesity (BMI 30.0–34.99 Kg/m^2^), class II obesity (BMI 35.0–39.99 Kg/m^2^) and class III obesity (BMI > 40.0 Kg/m^2^) [1, 2]. Besides, this associations vary across global regions and for younger and elder groups [1, 2].

Bioimpedance is a technique used to measure the electrical impedance or resistance of biological tissues or fluids. It involves the application of an electrical current, usually through the skin, and the measurement of the resulting voltage [3–5]. Bioimpedance is a non-invasive technique that can provide useful information on various physiological parameters, making it a valuable tool in healthcare and fitness settings. Several physiological parameters can be measured by bioimpedance, including body composition, hydration status, cell membrane integrity, tissue health among other.

The quantification of total body water (TBW), which includes both extracellular water (ECW) and intracellular water (ICW), plays a crucial role in diagnosing various health conditions [6–11]. For instance, dehydration can be identified through separate measurements of TBW and fat-free mass (FFM), whereas overhydration indicates the presence of oedema in individuals with heart disease or lymphoedema and mastectomy.

In the context of renal patients undergoing haemodialysis, a retention of fluid between treatments is observed. Assessing the volume of this excess fluid is vital to adjust the ultrafiltration process and understand how the fluid loss is divided between ECW and ICW [10, 11]. In patients on maintenance haemodialysis, recent studies observed an increase in the ECM/ICM index related with risk of sarcopenia. Bioimpedance technique is a non-invasive method to measure all of these anthropometric, metabolic and bioelectric parameters, offering a promising alternative to traditional techniques [3–12].

In recent years, the issue of overweight children and adolescents in Latin America and the Caribbean has become increasingly prevalent. Currently, an estimated 3 in 10 children and adolescents between 5 and 19 years old have overweight in the region [13, 14]. In 2020, UNICEF, The World Bank and WHO estimated that in Latin America and the Caribbean, a 7.5% of children under 5 years old, representing about 4 million children, are classified as overweight [13, 14]. This is higher than the global average of 5.7% [13, 14]. The origin of overweight and obesity statuses in childhood are the consumption of sugary drinks, ultra-processed foods and the lack of physical activity [13, 14].

In order to solve this issue, UNICEF has been promoting initiatives, in collaboration with governments from across the region, to improve the nutritional status of the population, guide families and communities, and contribute to regulatory actions to change food environments [13–17]. In addition, to avoid overweight, UNICEF supports nutritional campaigns in several countries and stimulates actions for the promotion, protection, and support of breastfeeding from birth to two years of age [13, 14]. Furthermore, scientists have created a US-Latin American research agenda on child obesity, finding evidence of anthropological factors influencing on the child obesity problem [13–17].

Alternatively, machine learning is a subfield of artificial intelligence that involves the development of algorithms and statistical models that enable computers to learn from data and make predictions or decisions without being directly programmed [18–20]. In medicine, machine learning has the potential to make more accurate diagnoses, decision making and personalized treatment plans [18–21]. Despite the development of bioimpedance method, there is no direct association between the bioelectrical parameters with weight status estimation. As the WHO system have the lack of variation between global regions and age for estimating the weight status, further studies are required to solve these problems. In this context, the aim of this work is to provide a predictive model, based on classification and regression learner methods, as a complementary approach to weigh status evaluation of younger volunteers from the main Oncological Hospital of Santiago de Cuba, Cuba.

## Methodology

We conducted a pilot random study at the Oncological Hospital in South Cuba, specifically in Santiago de Cuba. This is a public sector hospital specialized in cancer diseases. The study involved volunteers who were selected between 2002 and 2008 and aged between 2 and 18 years old. In total, 776 female and male volunteers were studied. This research followed the code of ethics, good medical and clinical practices established by the Health General Law of the Ministry of Public Health of Republic of Cuba (Number 41, 13 July 1983 and updated in 2010).

The research was evaluated and approved by the ethics committees and scientific councils of Provincial Blood Bank Renato Guitart, Oncological Hospital Conrado Benítez, Pediatric Hospital Juan Martinez Maceira and Pediatric Hospital Antonio María Béguez César. All relevant national regulations, institutional policies, and the Regional Committees for Medical, Health Research Ethics, and scientific council were in accordance with the tenets of the Helsinki Declaration. Additionally, parents of children and adult participants signed informed consent before starting the study.

The database collected in this study is not publicly available because it is still under study to extract more information that can provide valuable data for a better understanding of the behavior of bioelectrical parameters in healthy and diseased patients. However, the datasets are available from the corresponding author upon reasonable request.

Bioimpedance parameters were obtained by measuring using the standard tetrapolar whole-body configuration. The Bioimpedance analyzer used was the BioScan 98® model (Biológica Tecnología Médica S.L., Barcelona, Spain. URL: http://www.bl-biologica.es). Healthy volunteers participated in a previous fast for at least 3 h, with an empty bladder, and had not exercised or consumed alcohol in the previous 12 h. A 50 kHz frequency was used for the measurements. Adults used disposable pre-gelled Ag/AgCl electrodes model 3 M Red Dot 2560 (3 M, Ontario, Canada), while the paediatric sample used the 3 M 2248-50 Red Dot.

The study was performed in the morning by trained personnel in an air-conditioned room at 23 °C with an ambient humidity of 60–65%. To perform the measurement, subjects were placed in a supine position without clothing, without a pillow under their heads, with their arms separated 30° from the chest and their legs separated at an angle of 45° without contact between them, on a non-conductive surface. The electrodes were placed after cleaning the skin with 70% alcohol. The injector electrodes were placed medial to the dorsal surfaces of the hands and feet, close to the third metacarpal and metatarsophalangeal joints. Detector electrodes were placed between the distal epiphyses of the ulna and radius, at the level of the pisiform eminence, as well as at the midpoint between both malleolus respectively. The distance between the injector and detector electrodes was 5 cm.

To perform a serious AI study, machine learning involves problem formulation, dataset quality analysis, feature selection, and model generalization in the real world [18–24]. In this study, weight status is considered as the response, and bioelectrical and biological parameters of each volunteer constitute the features. With a large dataset of 393 individuals, we used a 95% cross-validation for training and 5% for validation to avoid the overfitting problem [23–25]. Initially, a feature selection of 24 features is made, followed by a down-selection under the simplest model premise for describing weight status. Various machine learning methods were used for classification and prediction of relevant bioelectrical and bioparameters. Further details will be provided along next sections.

## Results and discussion

### Weight status classification model using machine learning techniques

After revising the data, the cohort consisted of 393 infants and juveniles, including 184 females and 209 males. Within the cohort, 133 children are classified with normal weight, 128 overweight and 132 underweight based on age and sex. In this sense, the cohort is balanced according to sex and weight status. Other weight status classes were not encountered due to the age range. It is well-know that accessing to the weight status in preadolescents and early childhood is a problem that must deal with care due to the difference and the metabolic changes occurring during the growing [1, 2]. In this sense, we conducts a study in three age groups chronologically sored, namely 2 to 18 year old (2–18 age group compiling the entire cohort), 2 to 11 year old (2–11 age group) and12 to 18 year old (12–18 age group).

The weight status with classed normal weight, overweight and underweight is considered as the response for the classification model. Features considered for training include anthropometric parameters such as sex, height, weight, age, body surface area (BSA), body mass index (BMI), fat mass (MGC) according to Nhanes, total body water volume according to Kotler (TBW_Kotl_), skeletal muscle mass (smmbia), extracellular mass (ECM), body density (Densbia), fat-free mass according to Siri (FFM_S_) [26],. In addition, other bioelectrical parameters such as resistance (r), impedance (Z), capacitive reactance (Xc), phase angle (phase), corrected resistance (r_c_), corrected capacitance (x_c_) and specific resistivity (${\rho }_{00}$). To complete the features, other metabolic parameters are considered intra (ICW_Kotl_) and extracellular (ECW_Kotl_) water according to Kotler, extra-intracellular index (ECW_Kotl_/ ICW_Kotl_), basal energetic cost (GBE), and basal metabolic index (IMB).

**Fig. 1:**
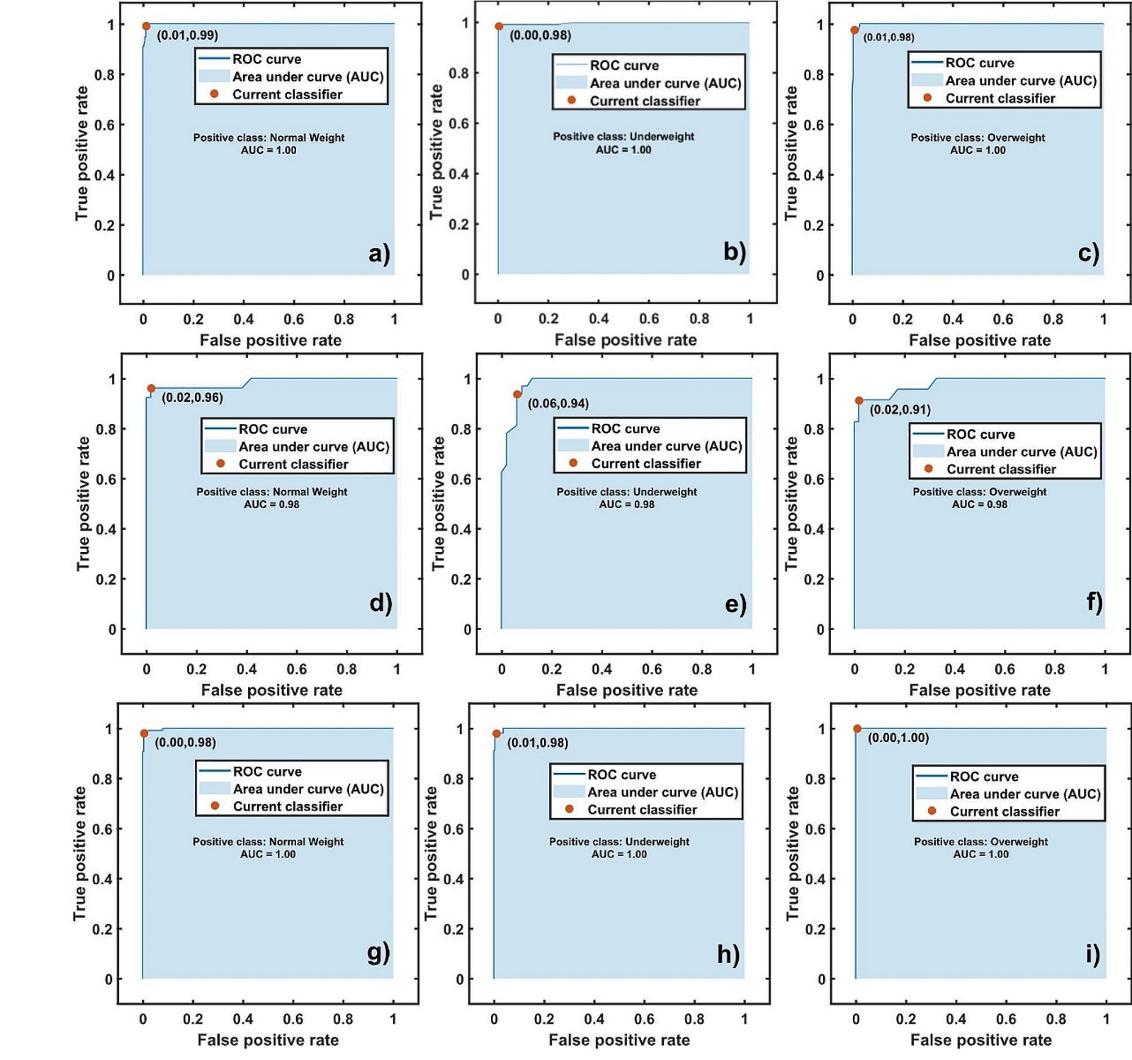
Receiver operating characteristic curve (ROC) for each positive class considered: **a)**, **b)** and **c)** corresponds to the complete cohort, **d)**, **e)** and **f)** to 2–11, while **g)**, **h)** and **i)** to 12–18 age group, respectively

The best classification model is the bagged trees ensemble for the three age group studied. For the entire cohort (i.e., 2–18 age group), the accuracy of the classification model is 98.50% and 5.00 total misclassification cost. For the 2–11 age group the accuracy is 93.80% with a total misclassification cost of 5.00, while for the 12–18 age group is 98.70% and 4.00 misclassification cost. The receiver operating characteristic curve (ROC) provides information regarding the true positive versus the false positive rate for a selected trained classifier [27–29]. The red point represents the values of the false negative rate (FNR) and the true positive rate (TPR) for the classifier. Furthermore, the area under the curve (AUC) provide information of the accuracy of the classifier model [27–29]. A larger AUC value indicates better classifier accuracy and predictions [27–29]. Figure 1 displays the ROC for each weight class and year group. From Figure a), b) and c) the AUC = 1.00, assigning low false positive rate observations incorrectly to the positive class in concern. For the 2–11 age group (Figure d, e and f), the classification model assigns 2.00% of the false positive rate observations incorrectly to the positive normal weight and overweight classes with an AUC of 0.98. Analogously, 6.00% of the false positive rate observations are wrongly assigned to the positive underweight class with AUC = 0.98. Analogously, for the 12–18 age group (Fig. 1g, h and i), the model predicts accurately the weigh status with AUC = 1.00 and low false positive rate of 1.00% for underweight individuals.

**Fig. 2:**
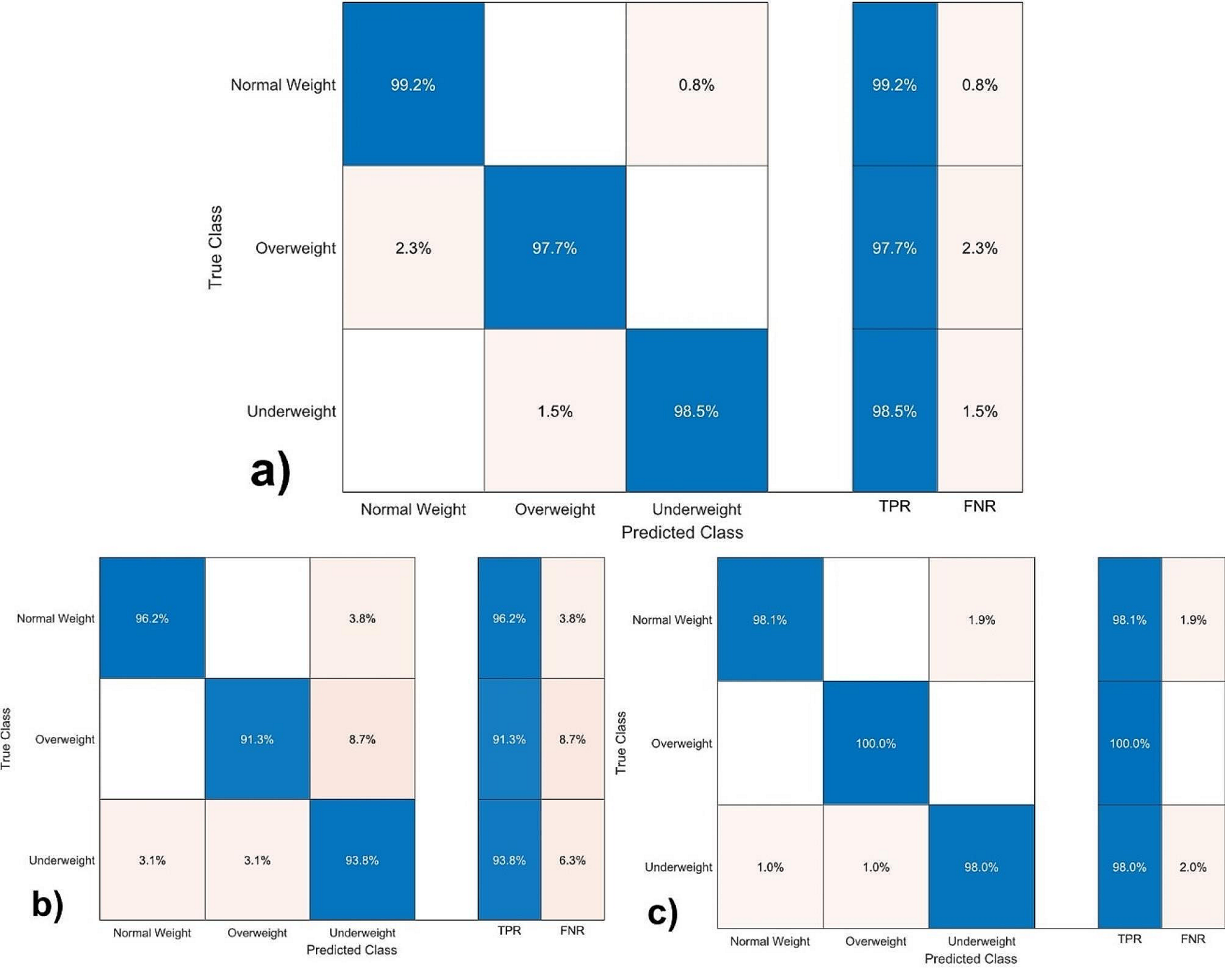
Confusion matrix for **(a)** 2–18, **(b)** 2–11 and **(c)** 12–18 age group, respectively

Confusion matrix provides detailed information about the accuracy predictions by comparing within specific classes [30, 31]. Figure 2 show the confusion matrix of each age group. For the entire age group (Fig. 2a) the model predict the normal weight status with an accuracy of 99.20%, the overweight and underweight statuses with a 97.70% and 98.50% of accuracy, respectively. In addition, the model misclassifies 0.80% underweight individuals as a normal weight, 2.30% of normal weight to overweight and 1.50% of overweight to the true class of underweight.

Analogously, for the younger age range (Fig. 2b), the model predicts the normal weight status with an accuracy of 96.20%, while the overweight and underweight with 91.30 and 93.81% of accuracy. However, the model assigns wrongly 3.80% to the normal weight class of underweight individual, 8.70% of underweight to overweight and 6.30% of normal and overweight to underweight class. From Fig. 2c, the overweight class is predicted with the highest accuracy. In addition, the normal weight and underweight classes are also predicted with a 98.00% of accuracy assigning 2.00% wrongly weight status classes to normal and underweight class. These findings can be explained considering that some individuals can be on a transition between weight statuses.

**Fig. 3:**
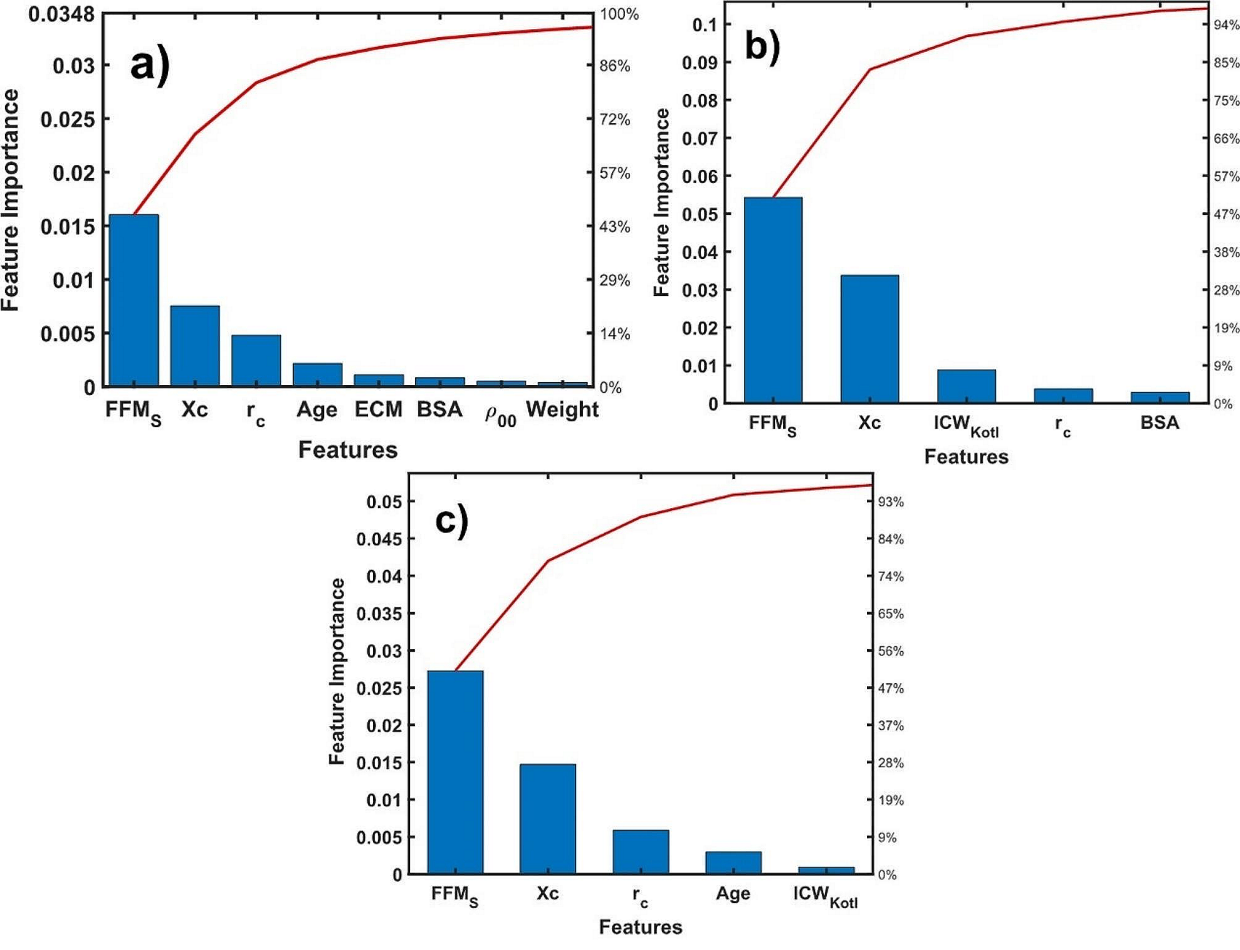
Feature importance of: **(a)** 2 to 18, **(b)** 2 to 11 and **(c)** 12 to 18 age group, respectively. The highest bar represents the most important characteristic of the classification model

Feature importance analysis is a critical step in the development and optimization of classification models, as it helps to identify the most relevant features and ensure the accuracy and reliability of the model, avoiding the common over fitting problems in regression learners [22–24]. Figure 3 displays the feature importance for each age groups. As it is shown in the Fig. 3a, the most important features of the entire age group are the fat-free mass (FFM_S_), reactance (Xc) and the corrected resistivity (r_c_). Among age groups, again the FFM_S_ and Xc prevails as the most important characteristics. Considering the other insignificant characteristics, the main difference is that the thirds important characteristic in the 2–11 age group (Fig. 3b) is ICW_Kotl_ and r_c_ in the 12–18 age group (Fig. 3c). Note that in the three cohorts the FFM_S,_ Xc and r_c_ play an important role accessing to the weight status of individuals.

The main results derived from the features importance analysis indicate that there are other characteristics derived from bioimpedance measurements that can be used alternatively for weight status predictions. For instance, the phase angle and the body density have more contribution to determine the weight status than the anthropometric body mass index (BMI). Reports concerning the failure of bioimpedance spectroscopy to determine the weight status have been described [32–34]. However, bioimpedance measurements are not superior to BMI as a predictor of overall adiposity in a general population [32, 33]. A study to determine obesity of 200 Taiwanese women with breast cancer by combining BIA and BMI revealed the underestimation of WHO criterion to state the cut-off of females with breast cancer [34]. On the other hand, both BIA and BMI methods can similarly detect normal and obese female individuals and are less accurate in determining underweight [32–34]. Our results suggest that there are other anthropometric and bioelectrical parameters (FFM_S_, Xc, r_c_) that can be used to support the diagnosis of weight status of pre- and adolescents individuals.

It is well-known FFM results in an important change of the human energy control of obese patients [35, 36]. Research shows that fat-free mass plays both an active and passive role in the body energy intake and requirements [13]. Increasing your fat-free body mass can be helpful for weight management [13]. The results supports those findings.

### The role of fundamental characteristics for accessing the weight status

**Fig. 4:**
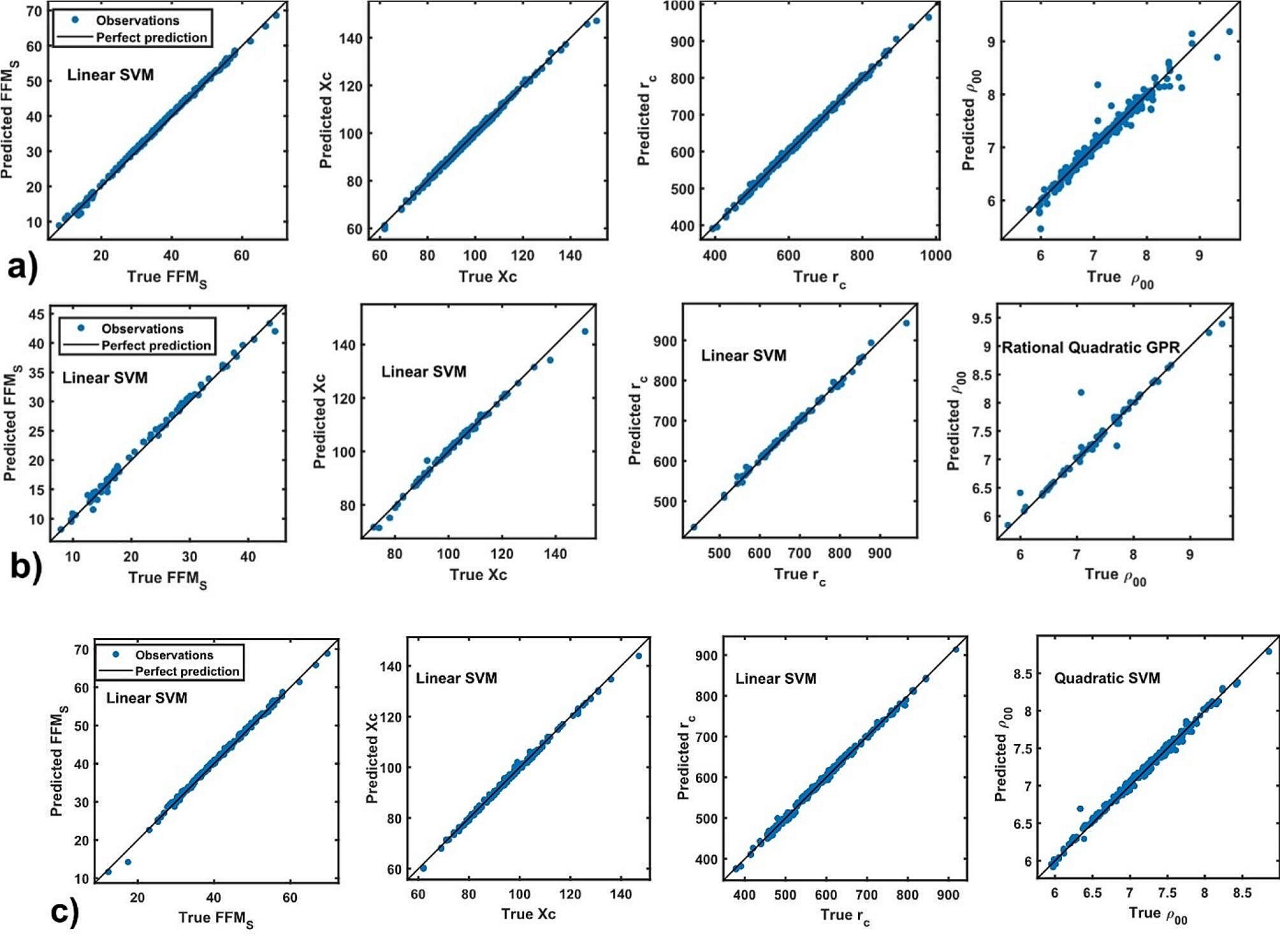
Response vs. predicted plot of the selected responses of each cohort: **(a)** 2–18, **(b)** 2–11 and **(c)** 12–18 age groups

Given that FFM_S_, Xc, and r_c_ are the primary characteristics that determine weight status in the studied cohort, this section focuses on developing regression models to predict these characteristics and assess their robustness in determining weight status. For the selected characteristic as the response, the data is divided into two parts: 95% for training and 5% for cross-validation. Figure 4a displays the predicted versus true response for the entire cohort, whereas Fig. 4b and c correspond to the 2–11 and 12–18 age groups, respectively. The observations are well-replicated by the chosen models, as indicated by all points lying near the control straight line. These results indicate good generalizability and high relevance of the identified features in explaining relative trends of the selected responses (FFM_S_, Xc, r_c_ and ${\rho }_{00}$).

Table 1 collects the results of the model for each response and their respective accuracy values. The accuracy is taken by considering the root mean square error (RMSE), R-squared (R^2^), mean square error (MSE) and the mean absolute error (MAE). As it is shown in Table 1, the main model describing these characteristic is the linear support vector machine (Linear SVM), except for ${\rho }_{00}$ where the best model is the rational quadratic (GPR) and quadratic support vector machine (Quadratic SVM) in the 2–11 and 12–18 age groups, respectively.

**Table 1:** Accuracy parameters of each model and age group categorized chronologically

Age Group	Response	Model	RMSE	*R* ^2^	MSE	MAE
Group 2–18	FFM_S_ (kg)	Linear SVM	0.59	1.00	0.35	0.49
	Xc (Ω)	Linear SVM	0.72	1.00	0.52	0.61
	r_c_ (Ω)	Linear SVM	5.27	1.00	27.81	4.20
	${\rho }_{00}$ (Ω)	Linear SVM	0.08	1.00	0.01	0.05
Group 2–11	FFM_S_ (kg)	Linear SVM	0.76	1.00	0.58	0.64
	Xc (Ω)	Linear SVM	1.21	1.00	1.47	0.74
	r_c_ (Ω)	Linear SVM	6.50	1.00	42.28	4.81
	${\rho }_{00}$ (Ω)	Rational Quadratic GPR	0.15	1.00	0.02	0.05
Group 12–18	FFM_S_ (kg)	Linear SVM	0.59	1.00	0.36	0.49
	Xc (Ω)	Linear SVM	0.72	1.00	0.51	0.56
	r_c_ (Ω)	Linear SVM	5.30	1.00	28.13	4.30
	${\rho }_{00}$ (Ω)	Quadratic SVM	0.01	1.00	0.05	0.05

**Fig. 5:**
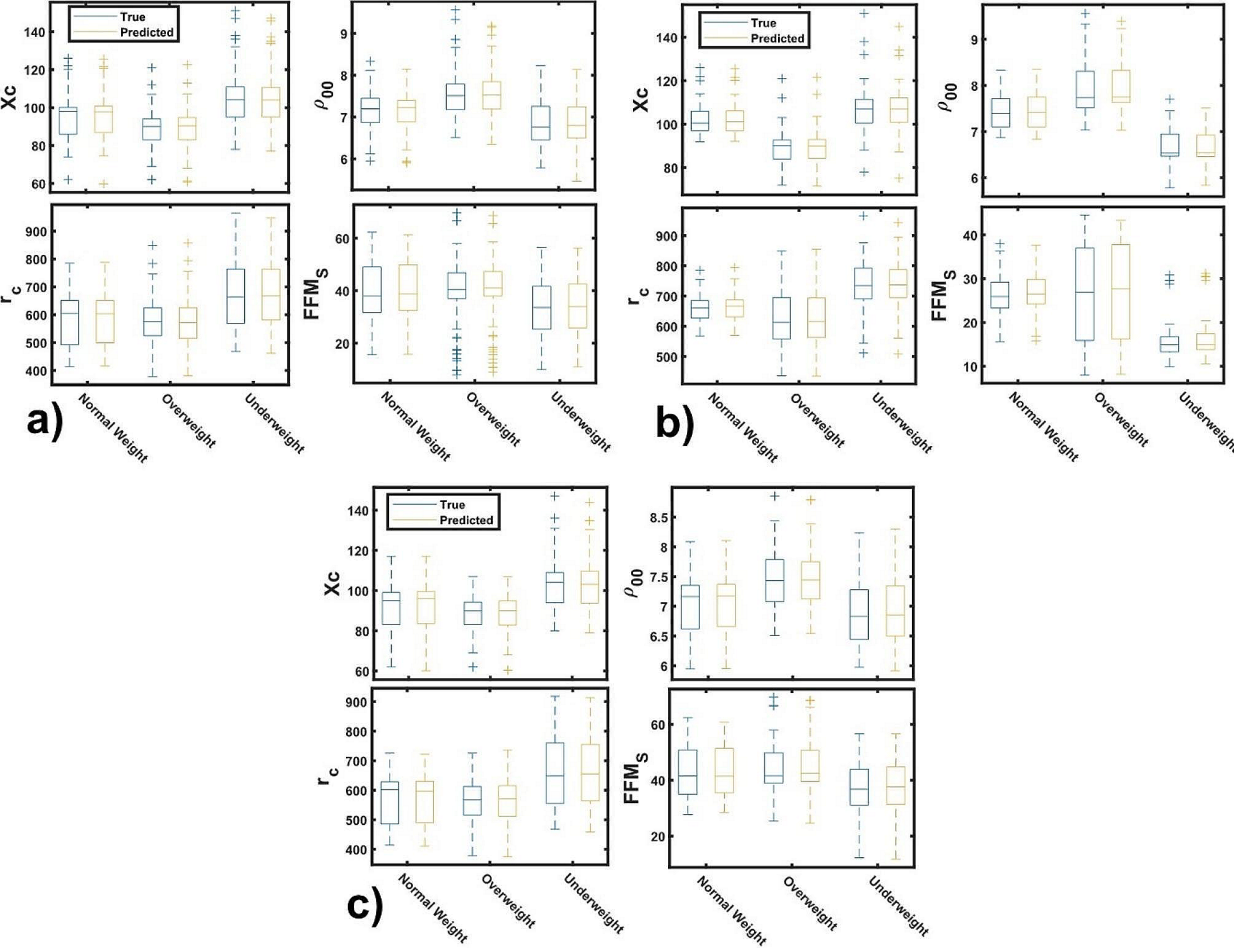
True and predicted anthropometric and bioelectrical parameters by weight status of each: **(a)** 2–18, **(b)** 2–11 and **(c)** 12–18 age group

Figure 5a shows the trend of response parameters in relation to the weight status of the entire cohort, while Fig. 5b and c display the results for the 2–11 and 12–18 age groups, respectively. Along the cohorts, underweight status have the lowest FFM_S_ and highest Xc and r_c,_ while the overweight have the lowest Xc and highest ${\rho }_{00}$. In the case of normal weight, the parameters lies between overweight and underweight statuses. These results agree with the affirmation that underweight individuals generally have larger phase angle because Xc is directly proportional to the phase angle, while overweight individuals have lower phase angle associated to the body fluid imbalance [35]. In our study the mean value of the phase angle are 7.07 ± 0.74º, 7.06 ± 0.45º and 6.70 ± 0.58º for underweight, normal and overweight status, respectively, of the entire cohort. In addition, the true and predicted values are close each other, reaffirming the accuracy of the models. In general, the predictor models reproduces well the observed parameters in all age groups. This finding supports the affirmation that by controlling the fat-free mass one can monitoring the fluid unbalance of individuals and the weight status as a consequence [36, 37].

**Fig. 6:**
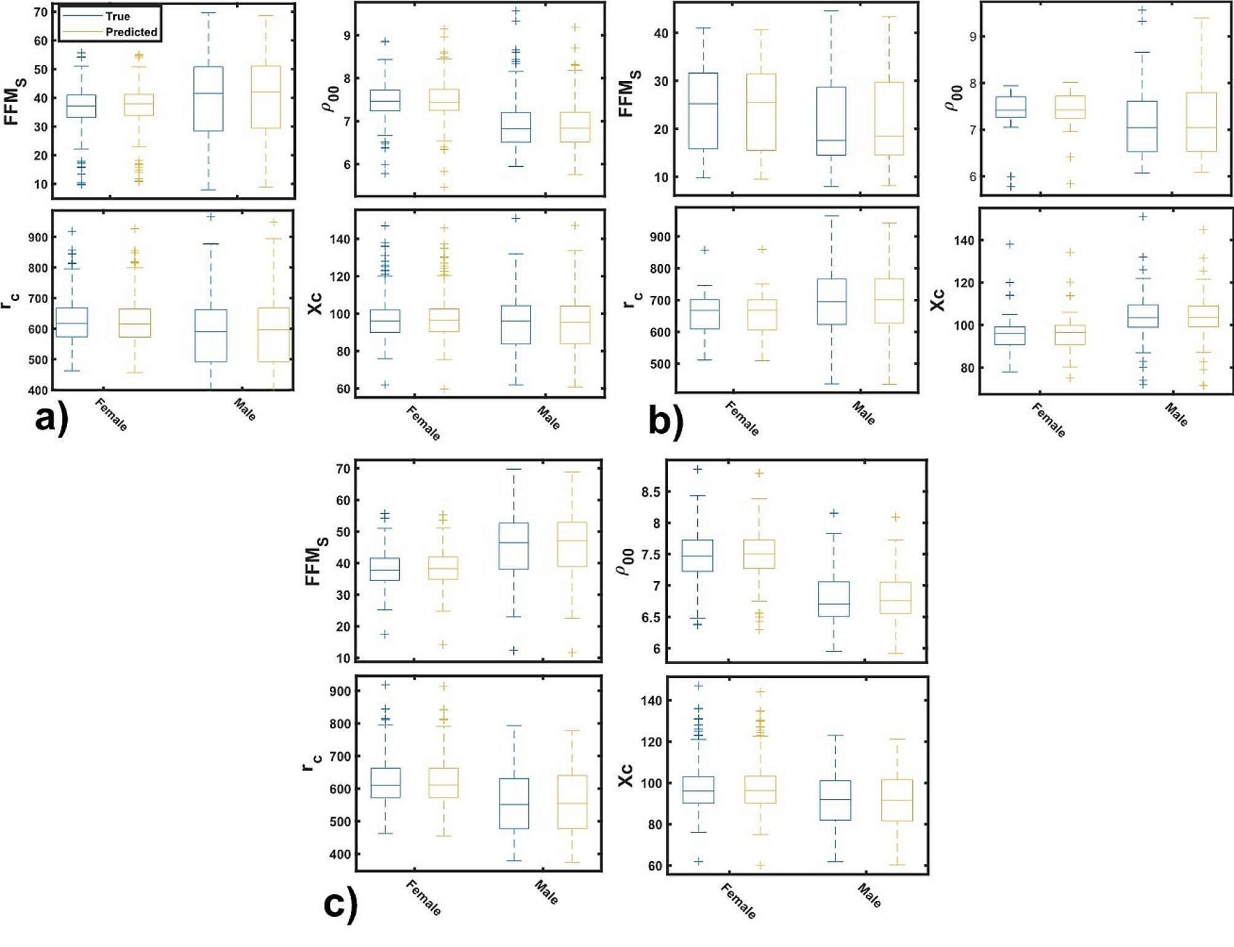
True and predicted anthropometric and bioelectrical parameters by gender class of: **(a)** 2–18, **(b)** 2–11 and **(c)** 12–18 age group

Figure 6 discloses the dependence of the above-mentioned responses upon gender of each age groups. In the entire cohort (Fig. 6a), there is no significant difference of r_c_ and Xc between sex, while boys have larger FFM_S_ and lower ${\rho }_{00}$ than girls. In contrast, for the early stage group (Fig. 6b), the difference is appreciated in all responses; boys have larger Xc and r_c_ than girls, larger FFM_S_ is observed for infant girls. For the adolescent group (Fig. 6c) the result shows that boys have larger FFM_S_ and lower r_c_, Xc and ${\rho }_{00}$.

The weight gain in children is primarily dependent on fat-free mass, fat mass in children tends to decrease during their development stages [38, 39]. Only after the onset of puberty and as sex differences in overall and regional body composition become more noticeable, there is a visible increase in body fat percentage [40, 41]. Once children hit puberty, sex hormones lead to changes in body composition. During puberty, young females typically experience an increase in body fat, particularly in the hips and breasts, while young males tend to see an increase in muscle mass [42].

## Conclusions

In this work, a predictive classification and regression learner model is used to study the association of weight status as a possible risk of disease of infant-juvenile cohort from Santiago de Cuba, Cuba. We used 24 characteristics derived from bioimpedance measurements, including other physical parameters. The classification model shows that there are other characteristics different than body mass index that can be used as a predictors of weight status [fat-free mass (FFM_S_), reactance (Xc), corrected resistance (r_c_) and specific resistivity (${\rho }_{00}$)]. The regression learner model was trained with the data and the abovementioned characteristics, predicting with high accuracy the weight status of the volunteers. The results concerning the variation of the above-mentioned characteristics against weight status and sex along the cohort agree with those reported in the literature. These predictive models developed in this work are of the great importance for accessing to the weigh status with high accuracy of younger individuals at the Oncological Hospital in Santiago de Cuba, Cuba.

## Data Availability

The datasets used during the current study available from the corresponding author on reasonable request.
